# Real-Time Tele-Mentored Low Cost “Point-of-Care US” in the Hands of Paediatricians in the Emergency Department: Diagnostic Accuracy Compared to Expert Radiologists

**DOI:** 10.1371/journal.pone.0164539

**Published:** 2016-10-17

**Authors:** Floriana Zennaro, Elena Neri, Federico Nappi, Daniele Grosso, Riccardo Triunfo, Francesco Cabras, Francesca Frexia, Stefania Norbedo, Pierpaolo Guastalla, Massino Gregori, Elisabetta Cattaruzzi, Daniela Sanabor, Egidio Barbi, Marzia Lazzerini

**Affiliations:** 1 Institute for Maternal and Child Health IRCCS "Burlo Garofolo”, Via dell’istria 65/1, Trieste, Italy; 2 University of Trieste, Piazzale Europa 1, Trieste Italy; 3 CRS4, Loc. Piscina Manna, Pula, Cagliari, Italy; Cambridge University, UNITED KINGDOM

## Abstract

**Background:**

The use of point-of-care ultrasonography (POC US) in paediatrics is increasing. This study investigated the diagnostic accuracy of POC US in children accessing the emergency department (ED) when performed by paediatricians under the remote guidance of radiologists (TELE POC).

**Methods:**

Children aged 0 to 18 years accessing the ED of a third level research hospital with eight possible clinical scenarios and without emergency/severity signs at the triage underwent three subsequent US tests: by a paediatrician guided remotely by a radiologist (TELE POC); by the same radiologist (UNBLIND RAD); by an independent blinded radiologist (BLIND RAD). Tele-radiology was implemented using low cost “commercial off-the-shelf” (COTS) equipment and open-source software. Data were prospectively collected on predefined templates.

**Results:**

Fifty-two children were enrolled, for a total of 170 ultrasound findings. Sensitivity, specificity, positive and negative predictive values of TELE POC were: 93.8, 99.7, 96.8, 99.4 when compared to UNBLIND RAD and 88.2, 99.7, 96.8, 98.7 when compared to BLIND RAD. The inter-observers agreement between the paediatricians and either the unblind or blind radiologist was excellent (k = 0.93). The mean duration of TELE POC was 6.3 minutes (95% CI 4.1 to 8.5). Technical difficulties occurred in two (3.8%) cases. Quality of the transmission was rated as fair, good, very good and excellent in 7.7%, 15.4%, 42.3% and 34.6% of cases respectively, while in no case was it rated as poor.

**Conclusions:**

POC US performed by paediatricians in ED guided via tele-radiology by an expert radiologist (TELE POC) produced reliable and timely diagnoses. Findings of this study, especially for the rarer conditions under evaluation, need further confirmation. Future research should investigate the overall benefits and the cost savings of using tele-ultrasound to perform US “at children’s bedsides”, under remote guidance of expert radiologists.

## Introduction

Point-of-care ultrasonography (POC US) is a bedside technology that enables clinicians to integrate clinical examination with real-time sonographic imaging [1–3]. In recent years emergency medicine physicians have increasingly adopted POC-US [1–3]. However, the advent of POC US has generated considerable controversy [3]: some believe that non-radiologists, if adequately trained, are able to achieve this practical competence and use it for the diagnosis of a number of different clinical conditions [3,4], while others believe that POC US is beyond the scope of busy physicians, and the skills and competences of a radiologist cannot be so easily acquired [3,5].

In the field of paediatric US, which is a specialised field of radiology, a recent review on POC US concluded that “limited research supports the notion that many POC US applications practiced by non-radiologists can assist in clinical decision-making and procedural success” [3]. However, evidence are rapidly accumulating. Several studies have evaluated ultrasound for the diagnosis of pneumonia in children [6–9], and POC US is increasingly used in this condition. Despite this is a very rapidly evolving field of medicine, so far only a few studies have evaluated other paediatric applications of POC US when in the hands of paediatricians. Overall, while the prospect of improving timely diagnoses is appealing, accuracy in the diagnosis and the final real benefits of POC US for children are still under evaluation [1–3,10]. Resistance to the use of POC US in the hands of “non radiologist” includes the fact that usually it addresses “yes” or “no” questions (e.g. presence or absence of fluid in the peritoneal cavity) and it can neither be compared with, nor substitute, a comprehensive evaluation conducted by a specialist radiologist [3,6]. Currently it is still unclear how many children, after a POC US performed by a non-radiologist, need to be evaluated by a radiologist as well [1–3,5,10]. Given all of the above considerations, the real benefit of POC US for children, their families, and for the health system is still unclear.

Tele-ultrasound is a branch of tele-medicine that has substantially improved over the last few years, driven by recent technological developments [11]. Tele-ultrasound has been used in some fields of medicine—such as adult medicine, pre-natal diagnosis and paediatric cardiology—for performing POC US under the guidance of an expert radiologist, who provided support and mentoring to the non-radiologist while performing the test [12–16]. Potential benefits of tele-point-of- care-ultrasound (TELE POC) include: increased access for patients to high quality care; performing more sophisticated and comprehensive examinations than would be possible in the hands of the non-radiologist alone; reducing the need for a radiologist’s evaluation after POC US tests; reducing waiting time and obviating transport of patients; better use of resources; possible cost savings and finally, provision of additional on-site training for non-radiologists in performing POC US.

This study was aimed at evaluating the diagnostic accuracy of POC US in the hands of paediatricians “at the patient’s bedside” in the emergency department (ED), under the remote support of paediatric radiologists (TELE POC). Diagnostic accuracy was assessed comparing US findings of paediatricians to US findings of specialist radiologists.

## Materials and Methods

### Study design

This was a prospective study on the diagnostic accuracy of POC US in the hands of paediatricians in the ED, under the remote support of paediatric radiologists (TELE POC). Diagnostic accuracy was assessed by comparing US findings of paediatricians to US performed by specialist radiologists.

The study is reported according to the STARD guidelines (Table A in S1 File) [17].

### Setting and population

The study was carried out at the Institute for Maternal and Child Health IRCCS Burlo Garofolo, a third level paediatric teaching hospital. Each year about 22,000 children access the paediatric ED, and out of these about 850 US examinations are performed.

Children aged 0 to 18 years referred to the paediatric ED in the period June 2013 to December 2013, during the working hours of the research team, with eight possible clinical scenarios—traumatic abdomen; suspected appendicitis; suspected intussusceptions; suspected hypertrophic pyloric stenosis; suspected pulmonary infection; unspecific abdominal pain; hip pain; soft tissue swelling—and without signs and symptoms of emergency or severity at triage were eligible for inclusion. Children were excluded from the study in the following circumstances: children identified by the standardised triage system as a “red code” or “yellow code” (i.e. emergency or severity); severe pain—defined as equal to or more than 8 on a Visual Analog Scale (VAS) from 0 to 10 or equivalent on age or Wong-Baker FACES^®^ Pain Rating Scale [18,19]; refusing participation.

The research team included four paediatric consultants working full time in the ED, and five paediatric radiologists, with over twenty years of experience in paediatric radiology. All the members of the research team were working on shifts, with the same working hours as other co-workers not participating in the study.

### Outcomes, study procedures, data collection

Diagnostic accuracy of POC US in the hands of paediatricians in the ED guided remotely by radiologists, was evaluated using as a gold standard the evaluation of the same child by senior paediatric radiologists (either blinded or unblinded to the POC US performed by the paediatrician).

In practice each enrolled child had to undergo three consecutive US examinations: a first US was performed by a paediatrician in the ED guided remotely via tele-ultrasound by a senior paediatric radiologist (TELE-POC); a second US was carried out by the same radiologist who guided the paediatrician (UNBLIND RAD); a third US was performed by a blind independent senior paediatric radiologist (BLIND RAD).

Three pre-defined comparisons of the US findings were set, based on who performed the US: 1) the paediatricians in the ED guided remotely by the radiologist (TELE-POC) versus the same radiologist who guided the paediatrician (UNBLIND RAD); 2) the paediatrician (TELE-POC) versus the blind radiologist (BLIND RAD); 3) the unblind radiologist (UNBLIND RAD) versus the blind independent radiologist (BLIND RAD). The first comparison aimed at evaluating whether the radiologist had reached the same diagnosis either by performing the test himself or via tele-ultrasound. The second comparison aimed at comparing results versus a blind expert (i.e. ruling out the possibility that lack of blindness could bias the un-blind radiologist), while the third comparison aimed at further evaluating differences in US findings among the blind and un-blind radiologists. The time interval between US performed by the paediatrician and the US performed by the radiologists could not exceed one hour.

As secondary outcomes, the study evaluated: time needed for performing the POC US in the hands of the paediatricians (in minutes); incidence of technical difficulties in performing tele-ultrasound (as incidence rate out of the total US); quality of the video and audio transmission via tele-ultrasound (rated by the radiologist receiving them using five grades: poor, fair, good, very good, excellent).

A pre-defined data extraction sheet was used for collecting prospectively the following information from each patient: date and time of the test, identification, age and sex of the child, quality of the US images, duration of the test performed via tele-ultrasound. A pre-defined checklist was used to collect data on the US findings (Table B in S1 File). Both the data collection sheet and the checklist were piloted before use. Data were transferred on the same day to an Excel database, without reporting any details that could disclose the identities of patients. Study procedures (Table C in S1 File) were summarised in a short guidance manual, available both in the ED and in the radiology department.

### Equipment

**Fig 1:**
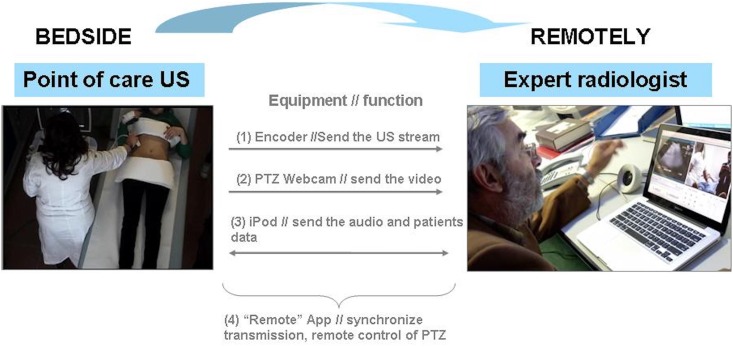
Components of the TELE-POC.

Fig 1 shows the components of the system. The tele-ultrasound system was built with low cost “commercial off-the-shelf” (COTS) equipment and open-source software. Specifically, the equipment procured for implementing tele-ultrasound consisted of: an encoder, a web-camera, an iPod with headphones, a USB microphone, and an open-source dual-licensed software system for real-timetele-ultrasound (Remote, CRS4; Remote Mobile App, CRS4 [20]). The hardware was procured as Commercial Off-The-Shelf (COTS), while the software was open-source [20]. Costs sustained for procuring all components of the tele-ultrasound equipment are reported in Table 1. The ultrasound machine in the radiology department and in the ED, together with the computer in the radiology department and the protected wi-fi connection were already available as standard equipment in the hospital.

**Table 1:** Technical specifications for the Tele-US equipment and costs sustained.

Equipment	COST [in euro]
**In the Emergency department**	
1. Network A/V Encoder	300
2. PTZ [pan/tilt/zoom] Web-camera [AXIS PTZ214]	1500
3. iPod [iPod Touch] with charger	185
4. Headphone with microphone for iPod [standard]	30
5. Software [*Remote*, CRS4; *Remote Mobile App*, CRS4]	free
**In the radiology department**	
6. USB Microphone in/out [standard]	30
**TOTAL**	**2045**

NOTE: Equipment already available included:

- Ultrasound machine in the emergency department: ESAOTE “MyLab40Advanced, using convex and linear probes as required by the different types of test

- Ultrasound machine in the radiology department: ESAOTE “MyLabClassC”, using convex and linear probes as required by the different types of test

- Computer in the radiology department: Apple Mac Book Pro, with OSXLion [10.7]

- Secure wi-fi connection between emergency department and radiology department

**Fig 2:**
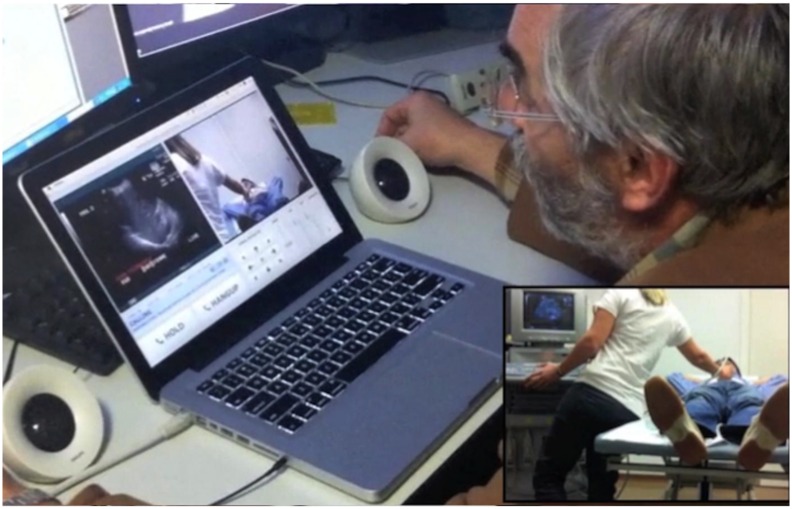
Images of the TELE-POC performed by the paediatrician in the emergency department (bottom right] as appears on the radiologist’s computer.

**Fig 3:**
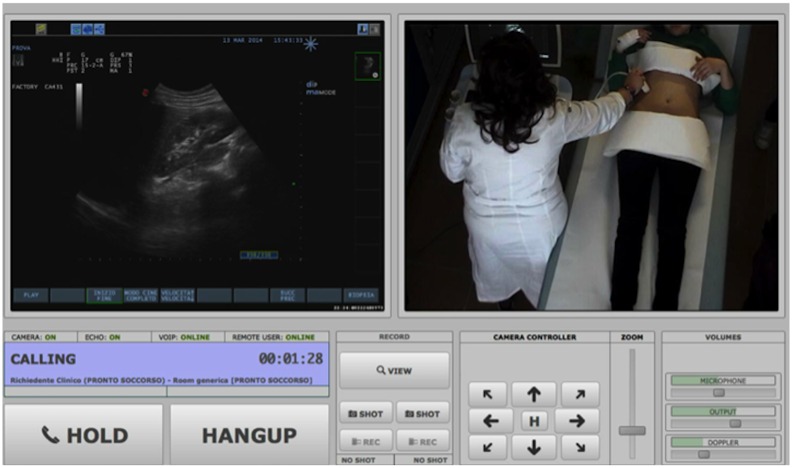
Detail of the images transmitted via tele-ultrasound, as appear on the radiologist’s screen, also showing the buttons for remote control of the webcam, zooming functions and volume.

In the ED, the encoder acquired the ultrasound stream from the ultrasound, while a robotised web-camera captured the images of the paediatrician performing the tests (Fig 2), and the iPod captured the audio signal. By means of the open-source software (Remote) the digitised ultrasound streams, together with the webcam images were synchronised and sent via the secure wi-fi line from the ED to the radiologist’s computer in the radiology department. The software [Remote] also allowed the radiologist to optimise by remote control the images received, by directing and zooming in/out the robotized PTZ (pan/tilt/zoom) webcam (Fig 3). An application installed in the iPod (Remote Mobile App) synchronised the audio signal, and was used to send the patient’s data from the paediatrician’s iPod to the radiologist’s laptop. Latency in sending the video and audio signal were pre-set to a maximum delay of 50 milliseconds.

The ultrasound units used in the ED and in the radiology department were comparable in terms of image quality, although the one used in the ED was a portable ultrasound unit.

### Training

A team of eight research paediatricians involved in the study underwent two individual training courses: a two-hour course was delivered by a senior paediatric radiologist (PG) on how to use the ultrasound machine for detecting the findings specified in the checklist (Table B in S1 File). Thirty minutes of training was delivered by the radiologist PI of this study (FZ) to each paediatrician involved on how to properly use all the equipment for tele-ultrasound in the ED.

### Statistical analysis

It was estimated that 170 US findings were needed to carry out the study, setting the level of the lowest acceptable sensitivity at 90%, the confidence interval around this level at 10% and the prevalence of positive evaluations in the study population at 20%.

Descriptive data are presented as mean, standard deviations (SD), median and 25%-75% interquartile range (IQR) for continuous variables, and as number and percentage for categorical variables. Categorical variables were compared using the two-sided chi-square, with a significance value of p<0.05.

The inter-observers agreement was evaluated with Cohen's K for the three pre-defined comparisons: 1) TELE-POC versus UNBLIND RAD; 2) TELE-POC versus BLIND RAD; 3) UNBLIND RAD versus BLIND RAD. A value of k.0.6–0.8 represents substantial agreement. For the same three comparisons, sensitivity, specificity, positive predictive value (PPV) and negative predictive value (NPV) were calculated, using a 2X2 table and adding 0.5 to any 0 value, for a more realistic and adjusted test performance. Results on sensitivity, specificity, PPV and NPV are reported as percentages with 95% confidence interval (95% CI).

To carry out this analysis, the US features predefined by the checklist were assumed to be independent: i.e. in cases of pulmonary US we considered as independent observations the evaluation of the presence of pleural effusion and of pulmonary consolidation.

In the case of effusions, the case was considered concordant if US findings had no more than 3 mm difference in pleural effusions, and 6 mm in Douglas effusions.

### Ethical considerations

The study was approved by the Independent Bioethical Committee of the Institute for Maternal and Child Health IRCCS Burlo Garofolo. Children and their families were informed about the study procedures, and written informed consent was obtained by all the children's parents or legal guardians. US images and patients’ details were sent from the ED to the radiology department using the hospital’s secure wi-fi connection. No details that could disclose the identity of the patients were collected in the database for data analysis.

The individual in this manuscript has given written informed consent (as outlined in PLOS consent form) to publish images of individuals (Figs 1 and 2).

## Results

**Fig 4:**
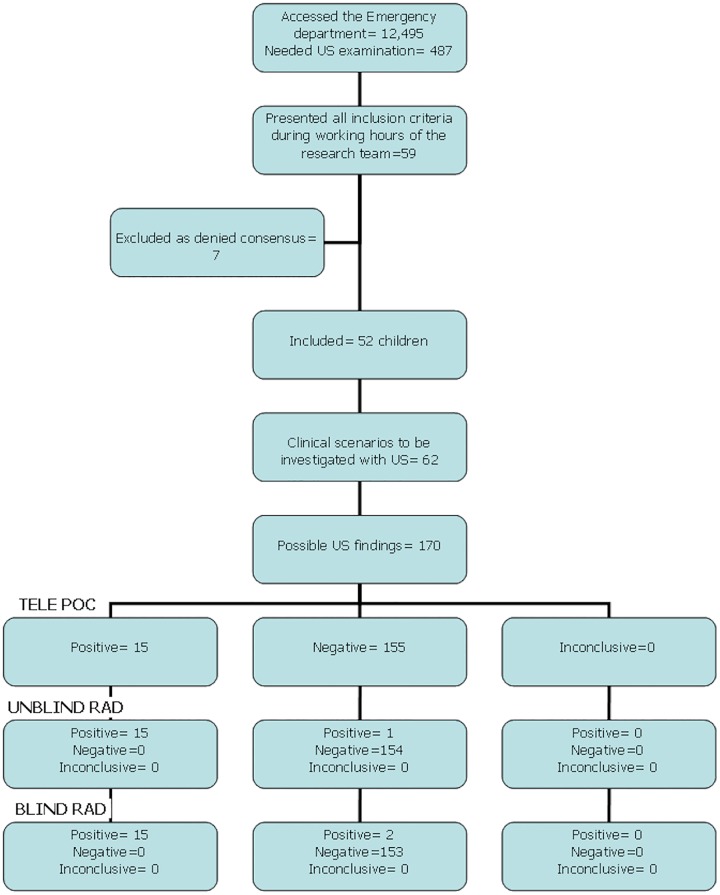
START Study flow diagram.

Throughout the research period, 59 children presented all criteria for inclusion. Of these, 52 (88.1%) children were included, while seven (11.8%) denied consent (Fig 4). All included children underwent the three expected US examinations. The time interval between US performed by the paediatrician, and the US performed by the radiologists never exceeded one hour.

Table 2 shows the characteristics of the children enrolled and the radiological findings observed by paediatricians. Out of the 52 children, eight (15.4%) were examined for two or more clinical presentations/suspects (e.g. typically suspected pulmonary infection and unspecific abdominal pain), for a total of 62 clinical presentations/suspects. According to the predefined study checklist (Table B in S1 File), the 62 clinical scenarios investigated corresponded to 170 possible US findings.

**Table 2:** Characteristic of enrolled children.

**General characteristics**	
Total children enrolled (N)	52
Sex	
- male	30 (57.7) ^§^
- female	22 (42.3)
Age—years	
- mean (95%CI)	8.1 (2.6–13.6)
- median (IQR)	7.0 (3.7–13.6)
**Clinical scenario**	**N (%)** ^§§^
Traumatic abdomen	32 (51.6)
Unspecific abdominal pain	11 (17.7)
Suspected appendicitis	3 (4.8)
Suspected intussusceptions	4 (6.4)
Suspected hypertrophic pyloric stenosis	1 (1.6)
Suspected pulmonary infection	7 (11.2)
Acute hip pain	3 (4.8)
Soft tissue swelling after trauma	1 (1.6)
**TOTAL**	**62 (100)** ^§§^

^§^ RR 0.73 (95% CI 0.49 to 1.09, p = 0.11)

^§§^ Eight children (15.4%) were examined for two or more suspected clinical conditions (e.g. suspected pulmonary infection and unspecific abdominal pain).

Out of the 170 possible US findings, paediatricians identified 155 cases as negative (91.1.%), and 15 (8.9%) as positive. With TELE-POC compared to both UNBLIND RAD or to BLIND RAD there were no false positive cases, while false negative occurred in one case (TELE-POC vs UNBLIND RAD) and two cases respectively (TELE-POC vs BLIND RAD) (Fig 4). Detailed results of the US findings are reported in Table 3.

**Table 3:** Detailed results of the US findings.

Ultrasound finding	N	Tele-POC		Unblind RAD		Blind RAD	
		Positive	Negative	Positive	Negative	Positive	Negative
Morrison effusion	29		29		29		29
Peri-hepatic effusion *	29		29	**1**	**28**	**1**	**28**
Peri-splenic effusion	28		28		28		28
Douglas effusion	31	3	28	3	28	3	28
Gall bladder litiasis	4		4		4		4
Hydronephrosis	7		7		7		7
Organomegalia	6		6		6		6
Distended bladder	6	1	5	1	5	1	5
Abdominal mass	5		5		5		5
Appendicitis	3	3		3		3	
Intussusceptions *	4		4		4	**1**	**3**
Hypertrophic pyloric stenosis	1	1		1		1	
Pleural effusion	7	4	3	4	3	4	3
Pulmonary consolidation	6	2	4	2	4	2	4
Intra-articular effusion	3	1	2	1	2	1	2
Hematoma	1		1		1		1
Total	170	15	155	16	154	17	153

* Discrepancies among the three groups are highlighted in bold

Sensitivity, specificity, PPV and NPV for the three comparisons are shown in Table 4. The inter-rather agreement for the evaluation of these items was excellent for each of the three comparisons (k = 0.93). False negative cases had the following characteristics: i) one was a case of a minimal per-hepatic effusion, identified by both the blind and un-blind expert radiologist; ii) the second case was an intermittent intussusception that was observed neither by the paediatrician nor the unblind radiologist, but only by the second radiologist. This latter case was labelled according to a conservative criterion as a false negative, although it could even have been a true negative (i.e. intussusceptions may have not been present at the time when the first tests were performed).

**Table 4:** Sensitivity, specificity, positive and negative predictive values.

	Sensitivity	Specificity	PPV	NPV
**TELE-POC vs UNBLIND RAD**	93.8 (71.7–98.9)	99.7 (97.0–100)	96.8 (75.3–99.7)	99.3 (96.4–99.4)
**TELE-POC vs BLIND RAD**	88.2 (65.7–96.7)	99.7 (97.0–100)	96.8 (75.3–99.7)	98.7 (95.4–99.6)
**UNBLIND RAD vs BLIND RAD**	94.1 (73.0–99.9)	99.7 (97.0–100)	97.0 (76.5–99.7)	99.4 (96.4–99.9)

Data reported as percentages (95% CI)

Abbreviations: NPV = Negative Predictive Value; PPV = Positive Predictive Value.

**Fig 5:**
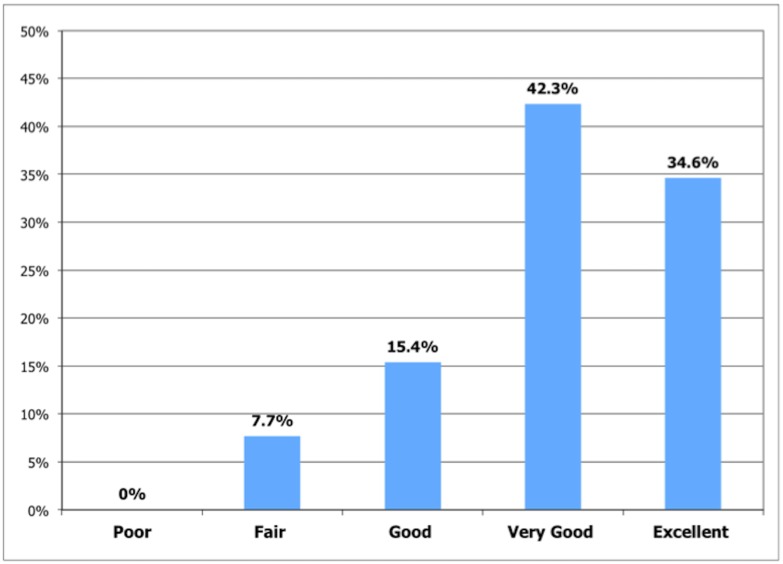
Quality of transmission via tele-POC.

The mean duration of the guided ultrasound session was 6.3 minutes (95% CI 4.1 to 8.5). Technical difficulties incurred in two (3.8%) cases: in one case there was a failure in the network, in a second case, occurring soon after the start of the study, the paediatrician was not able to properly set the connection for tele-ultrasound. Quality of the transmission via tele-ultrasound was rated as fair, good, very good and excellent respectively in 7.7%, 15.4%, 42.3% and 34.6% of cases, while in no case was it rated as poor (Fig 5).

## Discussion

Literature on tele-ultrasound for performing POC US in the paediatric ED is very limited. A previous study in Korea evaluated accuracy of tele-mentored US in diagnosing acute appendicitis in the ED, and reported that diagnostic accuracy was higher when emergency medicine residents were tele-mentored by experts emergency physicians, than for residents alone [21]. To be best of our knowledge, no other studies evaluated tele- US in the hands of paediatricians for different clinical scenarios in the paediatric ED. The study shows that diagnostic accuracy of POC US performed by paediatricians guided by an expert radiologist was extremely high when compared to the blind expert radiologist assessment [sensitivity 94.1%, specificity 100%; PPV 100%, NPV 99.3%]. The US examination only took a few minutes (mean time 6.3 minutes, 95% CI 4.1 to 8.5).

This study also highlights the feasibility of implementing tele-ultrasound with high quality standards (minimum delay in image transfer, high quality of transmission) at relatively low cost, using Commercial Off-The-Shelf (COTS) equipment, and open-source applications. This increases the potential transferability of results.

When looking at the transferability of the results of this study in other settings, the quality of the training delivered to paediatricians before implementing tele-ultrasound must be taken into account. Firstly, even though the training delivered to paediatrician involved for this project was relatively brief, it was highly focused and practical. Secondly, training was delivered by a paediatric radiologist with over 20 years of experience in training residents in paediatric US. Thirdly, although paediatricians had no previous experience on performing US, they were not completely “free from previous knowledge” since the study was conducted in a research institute where every week paediatricians attend a radiological meeting where clinical cases—including US images—are discussed. Additional feedback on how to better perform US and how to interpret US findings was provided during the tele-ultrasound sessions. When aiming at confirming the results of this study in other settings, local training needs should be considered carefully. Currently, there is limited consensus on the type of training needed for paediatricians to perform accurate POC US in children, and different scientific societies and international bodies have called for more research in this field [2,3,22]. Future studies could evaluate the performance of tele-mentored POC US when in the hands of other health professionals, such as for registrars in paediatric radiology, or for paediatricians in other settings [ambulatory care or others].

Clearly, in the field of POC US and tele-POC there are still many open questions. The more interesting questions, in our view, probably regard the evaluation of the overall benefits together with cost savings of using tele-POC to perform accurate and timely US “at children’s bedsides”, under the remote guidance of expert radiologists, operating from a remote location—including a remote hospital, city, country, or from home—and utilising different portable devices [23,24]. Despite the fact that data on cost-effectiveness are still limited [25], the available literature suggests potentially substantial cost-savings with tele-ultrasound [26,27]. For example, a preliminary cost-benefit analysis on real time tele-radiology in Sardinia, Italy, has shown a potential for reducing the costs of the system by 66% of the total expenditure, by cutting down on patient, transport costs and the cost of subsequent examinations [26]. A second study in Mississippi reported massive cost savings (US$ 1,126,683 vs. US$ 7,632,624, p < 0.001) after the implementation of telemedicine for managing trauma patients [27]. Future cost-effectiveness analyses should include both cost and benefits for children and their families (e.g. the cost of accessing the system, the time saved in receiving a final diagnosis and final health outcomes] and for the health system [e.g. the cost of having an expert available in the facility versus a tele-ultrasound service, cost of providing the test, cost-savings on patient transport etc).

Tele-ultrasound can also be used to support staff working in contexts with limited resources [28–32]. In addition to the experience reported in this paper, other studies have shown that the required technology for implementing effectively tele-ultrasound can now be procured at a relatively low cost, and its use is feasible and can be of benefit both for the staff and the population [28,31].

We acknowledge that children with emergency/severity conditions or with severe pain were not included in this study; this choice was necessarily made for ethical reasons, since it would not be ethical to perform three subsequent US for study purposes in children with emergency/severity conditions.

We acknowledge as a limitation of this study that, among the eight conditions that could be evaluated by POC US in the ED, the majority of cases were represented by abdominal trauma (51.6% of total cases) and unspecific abdominal pain (36.5%), while other conditions occurred in low numbers during the study period. We therefore recognise that our study should be considered as a pilot study on tele- POC in the paediatric ED, and its findings, especially for the rarer conditions under evaluation, need further confirmation. However, the study has the merit of investigating a novel approach. When compared to other existing studies in the paediatric field [3,10,33–38], our study represents a novelty, since no study so far has reported the use of tele-mentored POC US in the hands of paediatricians in the ED. Findings of this study suggest that tele-POC may be valuable for increasing the number of conditions evaluated by paediatricians with POC US in the ED. In the future, bigger studies would be needed to better document the benefit of different diagnostic approaches—such as POC US in the hands of paediatricians alone, versus POC US performed by paediatricians under remote expert guidance, versus POC US in the hands of an expert radiologist—for the diagnosis of all the different paediatric conditions included in this study.

### Conclusions

POC US performed by paediatricians in the ED guided via tele-ultrasound by an expert radiologist produced reliable and timely diagnoses. The results of this study, in particular for the rarer conditions under evaluation, need further confirmation. Future studies should evaluate the overall benefits and cost savings of using tele-ultrasound to perform US “at children’s bedsides”, under the remote guidance of an expert radiologist.

## Supporting Information

S1 File STARD Checklist (Table A); Predefined checklist used to collect US findings (Table B); Study procedures algorithm (Table C). (DOCX)
