# Supplementary material for: Real-Time Tele-Mentored Low Cost “Point-of-Care US” in the Hands of Paediatricians in the Emergency Department: Diagnostic Accuracy Compared to Expert Radiologists
Source: PLoS One. 2016 Oct 17;11(10):e0164539. doi: 10.1371/journal.pone.0164539 (PMC5066956; doi:10.1371/journal.pone.0164539)
Supplement: S1 File — (DOCX) [file pone.0164539.s001.docx]

**Table A. STARD Checklist**

| **Section and Topic** | **Item** |  | **On page** |
| --- | --- | --- | --- |
| TITLE/ABSTRACT/ KEYWORDS | 1 | [Identify the article as a study of diagnostic accuracy(recommend MeSH heading 'sensitivity and specificity').](http://www.stard-statement.org/item1_maintext.htm) | 1 |
| INTRODUCTION | 2 | [State the research questions or study aims, such as estimating diagnostic accuracy or comparing accuracy between tests or across participant groups.](http://www.stard-statement.org/item2_maintext.htm) | 5 |
| METHODS |  |  |  |
| *Participants* | 3 | [Describe the study population: The inclusion and exclusion criteria, setting and locations where the data were collected.](http://www.stard-statement.org/item3_maintext.htm) | 5 |
|  | 4 | [Describe participant recruitment: Was recruitment based on presenting symptoms, results from previous tests, or the fact that the participants had received the (evaluated) index tests or the (golden) reference standard?](http://www.stard-statement.org/item4_maintext.htm) | 5 |
|  | 5 | [Describe participant sampling: Was the study population a consecutive series of participants defined by the selection criteria in items 3 and 4? If not, specify how participants were further selected.](http://www.stard-statement.org/item5_maintext.htm) | 5 |
|  | 6 | [Describe data collection: Was data collection planned before the index test and reference standard were performed (prospective study) or after (retrospective study)?](http://www.stard-statement.org/item6_maintext.htm) | 6-7 |
| *Test methods* | 7 | [Describe the reference standard and its rationale.](http://www.stard-statement.org/item7_maintext.htm) | 6 |
|  | 8 | [Describe technical specifications of material and methods involved including how and when measurements were taken, and/or cite references for index tests and reference standard.](http://www.stard-statement.org/item8_maintext.htm) | 7-8 |
|  | 9 | [Describe definition of and rationale for the units, cut-offs and/or categories of the results of the index tests and the reference standard.](http://www.stard-statement.org/item9_maintext.htm) | 8 |
|  | 10 | [Describe the number, training and expertise of the persons executing and reading the index tests and the reference standard.](http://www.stard-statement.org/item10_maintext.htm) | 6 |
|  | 11 | [Describe whether or not the readers of the index tests and reference standard were blind (masked) to the results of the other test and describe any other clinical information available to the readers.](http://www.stard-statement.org/item11_maintext.htm) | 6 |
| *Statistical methods* | 12 | [Describe methods for calculating or comparing measures of diagnostic accuracy, and the statistical methods used to quantify uncertainty (e.g. 95% confidence intervals).](http://www.stard-statement.org/item12_maintext.htm) | 8 |
|  | 13 | [Describe methods for calculating test reproducibility, if done.](http://www.stard-statement.org/item13_maintext.htm) | 8 |
| RESULTS |  |  |  |
| *Participants* | 14 | [Report when study was done, including beginning and ending dates of recruitment.](http://www.stard-statement.org/item14_maintext.htm) | 5 |
|  | 15 | [Report clinical and demographic characteristics of the study population (e.g. age, sex, spectrum of presenting symptoms, co morbidity, current treatments, recruitment centers).](http://www.stard-statement.org/item15_maintext.htm) | 9,17 |
|  | 16 | [Report the number of participants satisfying the criteria for inclusion that did or did not undergo the index tests and/or the reference standard; describe why participants failed to receive either test (a flow diagram is strongly recommended).](http://www.stard-statement.org/item16_maintext.htm) | Fig 4 pag 22 |
| *Test results* | 17 | [Report time interval from the index tests to the reference standard, and any treatment administered between.](http://www.stard-statement.org/item17_maintext.htm) | 9 |
|  | 18 | [Report distribution of severity of disease (define criteria) in those with the target condition; other diagnoses in participants without the target condition.](http://www.stard-statement.org/item18_maintext.htm) | 9,17 |
|  | 19 | [Report a cross tabulation of the results of the index tests (including indeterminate and missing results) by the results of the reference standard; for continuous results, the distribution of the test results by the results of the reference standard.](http://www.stard-statement.org/item19_maintext.htm) | Fig 4, pag 22 |
|  | 20 | [Report any adverse events from performing the index tests or the reference standard.](http://www.stard-statement.org/item20_maintext.htm) | NA |
| *Estimates* | 21 | [Report estimates of diagnostic accuracy and measures of statistical uncertainty (e.g. 95% confidence intervals).](http://www.stard-statement.org/item21_maintext.htm) | 9,18 |
|  | 22 | [Report how indeterminate results, missing responses and outliers of the index tests were handled.](http://www.stard-statement.org/item22_maintext.htm) | 9,18 |
|  | 23 | [Report estimates of variability of diagnostic accuracy between subgroups of participants, readers or centers, if done.](http://www.stard-statement.org/item23_maintext.htm) | NA |
|  | 24 | [Report estimates of test reproducibility, if done.](http://www.stard-statement.org/item24_maintext.htm) | 9 |
| DISCUSSION | 25 | [Discuss the clinical applicability of the study findings.](http://www.stard-statement.org/item25_maintext.htm) | 10-11 |

**Table B. Predefined checklist used to collect US findings**

| **Clinical condition/suspected diagnosis** | **Ultrasound findings to be investigated** | **Check here** | **Characteristics** |
| --- | --- | --- | --- |
| **Traumatic abdomen** | 1) Morrison effusion | No □  Yes □ | If yes, mm ____ |
|  | 2) Peri-hepatic effusion | No □  Yes □ | If yes, mm ____ |
|  | 3) Peri-splenic effusion | No □  Yes □ | If yes, mm ____ |
|  | 4) Douglas effusion | No □  Yes □ | If yes, mm ____ |
| **Unspecific abdominal pain** | 1) Gall bladder litiasis | No □  Yes □ |  |
|  | 2) Hydronephrosis | No □  Yes □ | If yes, max diameter ____ |
|  | 3) Organomegalia | No □  Yes □ | If yes, max diameter ____ |
|  | 4) Distended bladder | No □  Yes □ |  |
|  | 5) Abdominal mass | No □  Yes □ | If yes, max diameter ____ |
| **Suspected pulmonary infection** | 1) Pleural effusion | No □  Yes □ | If yes, mm ____  If yes, corpuscular No □ Yes □ |
|  | 2) Pulmonary consolidation | No □  Yes □ |  |
| **Suspected appendicitis** | Appendicitis | No □  Yes □ | If yes, max diameter ____ |
| **Suspected intussusceptions** | Intussusceptions | No □  Yes □ |  |
| **Suspected hypertrophic pyloric stenosis** | Hypertrophic pyloric stenosis | No □  Yes □ |  |
| **Acute hip pain** | Intra-articular effusion | No □  Yes □ | If yes, mm ____ |
| **Soft tissue swelling after trauma** | Hematoma | No □  Yes □ | If yes, max diameter ____ |

**Table C. Study procedures algorithm**

| 1. The child arrives in the ED is triaged   **↓**   1. The paediatrician evaluates if inclusion criteria are present, exclusion criteria are absent   **↓**   1. The paediatrician explains the study objectives and informs the child and his/her family about the study procedures   **↓**   1. The paediatrician and the family fill in the consent form   **↓**   1. The paediatrician contacts the radiology department that is in this way alerted   **↓**   1. The paediatrician collects the child’s demographic data and sends them to the radiology department   **↓**   1. The paediatrician performs the US in the ED under the guidance of the unblind radiologist and fills in the data collection template #1   **↓**   1. The patient is immediately sent to the radiology department without disclosing any results of the US performed in the ED   **↓**   1. The “unblind” radiologist performs the US and fills in the data collection template #2   **↓**   1. The “unblind” radiologist immediately refers the child to the “blind radiologist” without disclosing any results of Us examination   **↓**   1. The “blind” radiologist performs the US and fills in the data collection template #3   **↓**   1. The “blind” radiologist fills in the data collection template #3 |
| --- |
